# Oligometastatic Adenocarcinoma of the Esophagus: Current Understanding, Diagnosis, and Therapeutic Strategies

**DOI:** 10.3390/cancers13174352

**Published:** 2021-08-28

**Authors:** Michael P. Rogers, Anthony J. DeSantis, Christopher G. DuCoin

**Affiliations:** 1 Department of Surgery, Morsani College of Medicine, University of South Florida, Tampa, FL 33606, USA; mrogers6@usf.edu (M.P.R.); adesanti@usf.edu (A.J.D.); 2 Department of Surgery, Division of Gastrointestinal Surgery, Morsani College of Medicine, University of South Florida, Tampa, FL 33606, USA

**Keywords:** esophageal adenocarcinoma, esophageal cancer, oligometastasis

## Abstract

**Simple Summary:**

The diagnosis and management of oligometastatic esophageal adenocarcinoma remains nuanced. Early diagnosis may allow for prompt intervention and, ideally, prolonged patient survival. As recent and emerging trials shed new light on this topic, we sought to identify the current understanding and treatment recommendations for oligometastatic disease by performing a thorough review of the available literature.

**Abstract:**

Esophageal adenocarcinoma is an aggressive cancer of increasing incidence and is associated with poor prognosis. The early recognition of synchronous and metachronous oligometastasis in esophageal adenocarcinoma may allow for prompt intervention and potentially improved survival. However, curative approaches to oligometastatic esophageal disease remain unproven and may represent an area of emerging divergence of opinion for surgical and medical oncologists. We sought to identify the current understanding and evidence for management of oligometastatic esophageal adenocarcinoma by performing a thorough review of the available literature.

## 1. Introduction

Esophageal adenocarcinoma (EA) comprises up to 80% of esophageal cancer in the United States and is a major cause of cancer morbidity and mortality worldwide [1]. While esophageal squamous cell carcinoma has been declining in the United States and in other parts of the western world, EA incidence has experienced a five-fold increase over the last four decades [2]. The five-year overall survival rate from 2009–2015 was 19.9%, with patients without lymph node involvement experiencing significantly better prognosis than those with involved lymph nodes [3]. Although surgical resection remains the mainstay treatment, survival is poor due to the high incidence of locoregional or distant metastasis [4,5,6]. Accordingly, a multimodal approach of neoadjuvant chemoradiotherapy (i.e., cT1b-T4a, N0-N+ staged patients) or perioperative chemotherapy alone (i.e., cT4b staged patients) has become the standard of care [7,8]. While oncologic outcomes have improved with multimodal therapy, five-year survival rates continue to give cancer specialists pause. This poor prognosis has highlighted the need for refinements in current treatment practices and pursuits of novel understandings in tumor biology, microenvironment, invasion tactics, and metastatic potential. The early identification and treatment of oligometastatic esophageal adenocarcinoma represents one such strategy.

Though no exact definition exists, oligometastatic disease is generally characterized by a state of limited metastatic burden of less than five metastases, and can be detected at the time of primary cancer diagnosis, so-called synchronous oligometastasis, or detected following treatment of the primary tumor, known as metachronous oligometastasis [9,10]. Whether this state represents an intermediate step in widespread metastasis or is a distinct molecular metastatic pattern remains debatable [11]. Regardless, the potential for improved survival outcomes with early detection, and thus earlier treatment, is of significant interest. Metastatic esophageal disease is largely regarded as an end-stage condition, with many patients offered palliative therapy. Consequently, the ability to detect and intervene prior to widespread metastatic burden remains a topic of significant research. Currently, no consensus treatment guidelines exist for oligometastatic esophageal cancer, mainly due to the paucity of large randomized control trial data in this cohort. We sought to identify the current understanding, diagnostic tools, and treatment modalities available in oligometastatic esophageal adenocarcinoma by performing a thorough review of the available literature.

## 2. Methods

**Figure 1:**
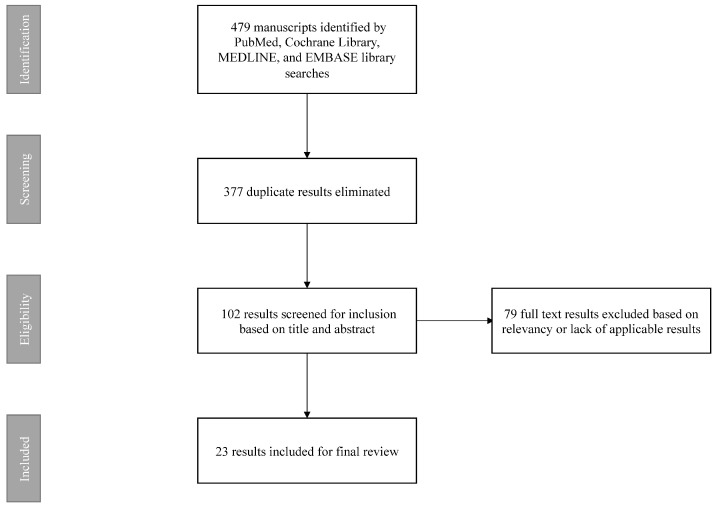
Flow chart of selection strategy for included reviewed manuscripts following PRISMA guidelines.

A comprehensive review of the available literature was performed on 30 May 2021, using PubMed, Cochrane Library, MEDLINE, and EMBASE databases. Search terms included the following: “oligometastatic esophageal adenocarcinoma”, “oligometastasis”, “esophageal cancer oligometastasis”, “esophageal adenocarcinoma oligometastasis”, “oligometastatic gastroesophageal adenocarcinoma”, and “oligometastatic esophageal cancer”. Articles were limited to those published in English and German. Given the relative scarcity of available literature, search results’ references were thoroughly reviewed for possible inclusion to ensure the maximal amount of available information was captured. Available results were manually reviewed thoroughly for relevance and included retrospective observational studies, prospective multicenter trials, an ongoing prospective randomized trial, and a systematic review of the available literature. Duplicate results and those unrelated to the subject matter were eliminated from further review. Though not a systematic review, screening and eligibility for inclusion of relevant studies followed standard PRISMA guidelines (Figure 1).

## 3. Discussion

### 3.1. Diagnostic Approaches

General diagnostic approaches to esophageal carcinoma with suspected oligometastases follow the traditional work-up strategy when staging esophageal cancer. Depth of tumor invasion and nodal involvement are the best predictors of long-term survival and an important determinant of therapeutic approach, making thorough initial staging essential to optimize patient outcome. Endoscopy and tissue biopsy remain the initial steps, with careful documentation of tumor location, length, extent of circumferential involvement, and presence of associated Barrett’s esophagus of vital importance [12]. Additionally, endoscopic ultrasound (EUS) is generally recommended to aid in assessing tumor depth and nodal staging. The diagnostic yield is increased when EUS is combined with fine-needle aspiration (FNA) when evaluating lymph node metastasis [13]. The potential presence of synchronous or metachronous double primary malignancies also highlights the importance of definitive pathologic tissue diagnosis, as the existence of a second distinct tumor will ultimately change treatment strategy (i.e., esophageal adenocarcinoma with synchronous primary lung adenocarcinoma) [14]. Additionally, careful diagnostic planning for tissue diagnosis should be considered in patients who have previously undergone tumor resection and present with metachronous metastases, as former procedures may alter the diagnostic and treatment approach. Cross-sectional imaging with contrast enhanced computed tomography (CT) of the chest and abdomen is recommended for the staging of locoregional and evaluating potential metastatic disease. Recently, fused positron emission tomography-CT (PET-CT) has improved diagnostic ability by merging anatomy and metabolic data and has emerged as the preferred imaging modality in patients with advanced locoregional disease for both initial imaging and to determine response of neoadjuvant therapy [15].

Studies evaluating the diagnostic approaches to synchronous and metachronous oligometastatic esophageal disease have included the aforementioned modalities; however, follow-up regimens were not generally specified to assess variations in practice and the impacts on early detection of disease [9]. Current guidelines recommend post-surgical imaging with contrasted CT be considered every 12 months for 3 years if additional curative-intent therapy for recurrence is likely in patients with small tumors with nodal disease [16]. For larger tumors following trimodal therapy, CT with contrast should be considered every 6 months for up to 2 years if additional curative-intent therapy for recurrence is likely [16]. No guidelines currently exist for the evaluation of oligometastatic disease specifically, likely due to the paucity of data in this small cohort. Symptomatic patients should be investigated with prompt imaging and may be guided by presentation of symptoms (i.e., brain magnetic resonance imaging (MRI) in the presence of neurologic symptoms).

### 3.2. Molecular Mechanisms

Important insights have been made into the molecular underpinnings of esophageal adenocarcinoma and their role in oligometastatic and widely metastatic disease. Various studies have identified potential mechanisms contributing to increased tumor size, invasion, and metastasis. These pathways have improved the understanding of esophageal carcinoma and identified potential novel therapeutic targets. Although the precise molecular mechanisms contributing to oligometastatic disease remains elusive, several potential contributors of invasion and metastatic spread have been identified and are the focus of ongoing investigations.

Somatic point mutations in the tumor suppressor TP53 (responsible for p53 protein production) represent the most frequent gene mutations occurring in approximately 50% of esophageal carcinomas [17]. Efforts at exome and whole-genome sequencing have identified a high frequency of mutations in esophageal carcinoma, outpaced by only melanoma and lung cancer [17,18]. Other significantly altered genes, including p16/CDKN2A, ELMO1, DOCK2, ARID1A, SMARCA4, and ARID2, have been implicated in metastatic potential through various mechanisms [17].

Wang et al. recognized increased lymph node metastases in esophageal adenocarcinoma specimens that over-expressed Dickkopf-3 (DKK3) [19]. A member of the Wnt inhibitor family, evidence suggests DKK3 may act as a tumor suppressor in the metastatic setting in some cancers and is over-expressed, leading to cancer invasion, angiogenesis, and chemoresistance in others. DKK3 has been hypothesized to regulate FGF and Activin/Nodal via SMAD4 and influence the TGF-β pathway. The authors demonstrated significant over-expression of DKK3 in esophageal adenocarcinoma, promoting increased proliferation, invasion, and chemoresistance, and they suggested it may play an important role in tumor growth and metastatic disease [19]. Toll-like receptor 5 (TLR5) and tumor-associated glycoprotein 72 (TAG-72) have also been implicated in lymph node metastases [20,21]. Xu et al. found TAG-72 levels significantly correlated with lymph node status and the extent of invaded lymph nodes, suggesting its use as a potential future clinical predictor [21]. TLR5 has been proposed to activate nuclear factor-κB (NF-κB) in gastric cancer [22,23]. Suppression of NF-κB in gastroesophageal junction carcinoma cell lines leads to a blockade of metastasis, and is thus felt to be implicated in metastatic potential when activated [22].

IGF2 mRNA binding protein 2 (IGF2BP2/IMP2) was originally identified as an autoantigen in hepatocellular carcinoma but has also recently been implicated in esophageal adenocarcinoma [24]. Barghash et al. demonstrated esophageal adenocarcinoma, and Barrett’s esophagus tissue showed over-expression of IMP2, particularly in those of increased size and in metastatic tissue. IMP2 is involved in cell metabolism and high expression correlated with growth, proliferation, metabolism, inflammation, and cancerous processes [24]. IMP2 expression leads to elevated levels of IGF2, which may activate MAPK and Jak-STAT signaling pathways and is associated with poor prognosis in esophageal carcinoma [22].

Various single case reports of alpha-fetoprotein (AFP) producing esophageal adenocarcinomas leading to liver metastases are described in the literature, but have not been described in large cohorts [25,26,27]. Aside from the aforementioned contributors to lymph node metastases and sporadic case reports, existing literature is sparse concerning the precise underlying mechanisms contributing to oligometastatic disease.

While the complete cadre of contributing molecular factors has yet to be elucidated, important inroads into understanding these processes has been made. As additional investigations continue to improve our understanding of the genes and molecular mechanisms involved in oligometastatic EA, targeted therapies may improve the relatively discouraging five-year survival rates currently experienced.

### 3.3. Current Management

Current treatment strategies in the United States for esophageal adenocarcinoma rely on the recommendations of the National Comprehensive Cancer Network (NCCN, Plymouth Meeting, PA, USA) guidelines and are generally based on a multidisciplinary team approach tailored to the individual patient’s American Joint Committee on Cancer (AJCC, Chicago, IL, USA) stage, Siewert–Stein classification, co-morbidities, and other factors [28]. In patients with locally advanced (T3-T4) or cN1-N3 (lymph node metastasis according to clinical evaluation) esophageal tumors, neoadjuvant chemotherapy or chemoradiotherapy plus resection is required, with most centers tailoring this approach based on histologic subtype [7,16,29]. Traditional dogma and guidelines recommend against attempted curative resection and metastasectomy in patients with cancers that are felt to be unresectable, or in those with distant disease (T4b, any N, or M1), with instead a focus on palliative chemoradiotherapy [29,30]. However, contemporary literature has somewhat challenged this philosophy. The recent results of the multicenter German AIO-FLOT3 and AIO-FLOT4 studies evaluating locally advanced, resectable tumors of the esophagogastric junction (EGJ) and stomach suggests well-selected patients may benefit from surgery and peri-operative chemotherapy, and indeed has provided rational for further randomized clinical trials in this cohort [31,32]. These included patients with histologically confirmed, previously untreated, nonmetastatic, operable (>T2, N any, and M0 or any T, N+, and M0), or metastatic (T any, N any, and M1) adenocarcinoma of the stomach or gastroesophageal junction without disease recurrence or uncontrolled medical illness, and with sufficient bone marrow and kidney function [31]. Additional investigations from subgroup analyses of clinical trials, retrospective patient cohorts, the Japan Clinical Oncology Study, and current RENAISSANCE (AIO-FLOT5) trial also highlight the ongoing debate of surgical intervention in limited metastatic gastric and esophagogastric cancers [33,34].

### 3.4. Evidence for Management of Oligometastasis

Evidence for management of oligometastasis is relatively limited, but primarily encompasses retrospective observational studies and emerging prospective trial data analyzing resection of pulmonary metastasis, liver metastasis, or multiple oligometastatic sites [35,36,37,38,39,40,41,42,43]. The relatively few cases of primary esophageal adenocarcinoma included in these studies makes this evidence even more nuanced. Meta-analysis by Jamel et al. demonstrated the majority of these studies involve primarily squamous cell carcinoma of the esophagus, with adenocarcinoma representing only 23% of histologic subtypes evaluated [9]. Schizas et al.’s more recent systematic review identified 420 patients from six studies that primarily included adenocarcinoma (77.3%), followed by squamous cell carcinoma (22.7%) [44]. The variation of subtypes in these studies likely highlights the paucity of available evidence and the increasing incidence of adenocarcinoma in this cohort. Additionally, the treatment management between synchronous and metachronous oligometastasis is distinct in approach. Nevertheless, important clinical insights may be gleaned from these reports.

Consideration of various treatment aspects should be weighed prior to embarking on aggressive treatment modalities: is curative resection possible, will quality of life change or improve, will overall survival improve, can complications from resection be mitigated, and is overall cure a possibility [45]? Ideally, surgical resection of the primary tumor and all metastases should be possible when presenting with synchronous disease. Standard resection strategies stratified by localization of the primary tumor with lymphadenectomy of regional and abdominal lymph nodes is recommended. Cervical lymphadenectomy should also be considered for cervical esophageal tumors [46]. Schmidt et al. retrospectively evaluated 123 patients with metastatic gastric and esophageal carcinomas (70 patients with adenocarcinoma of the EGJ, 53 patients with gastric cancer), of which 112 underwent resection and 72 patients received neoadjuvant chemotherapy [41]. An R0 resection was achieved in 63 patients, including metastasectomy. Patients presented with various metastatic sites: 38% with distant lymph node, 24% liver, 14% peritoneal, and 9% lung metastases. Resected patients had a median overall survival of 21.3 months, with complete resection and pre-operative chemotherapy prolonging survival to a median of 29.5 months [41]. In multivariate Cox regression analysis, site of metastasis, including distant lymph node metastases, did not appear to influence survival; however, prognosis was strongly influenced by neoadjuvant chemotherapy. In the context of metachronous presentation, recent work by Ghaly et al. to identify prognostic factors for survival in a cohort of 56 patients following multimodal treatment of metachronous oligometastasis did not demonstrate significant survival differences between groups based on metastatic location [36]. The time to recurrence in this series was found to be a significant predictor of survival, with those presenting with recurrence within 12 months of treatment having worse survival. These findings are in agreement with other investigations into disease-free survival time and the effect on long term survival in other cancers.

In contrast, other retrospective cohort studies have found the site of metastasis to affect prognosis. Ichida et al.’s 2013 retrospective analysis of survival following resection of liver and lung metastases showed those with pulmonary recurrences had superior outcomes (median survival of 13 months) compared to those with recurrences of the liver (median survival of 5 months) or other sites (median survival of 3 months) [42]. Surgical resection of pulmonary metastasis conferred survival benefit over a non-resection approach (median survival of 48 months vs. 10 months) [42]. However, hepatic metastasectomy did not show significant survival benefit in this cohort. Additional small series, including Hiyoshi et al. and Huddy et al., demonstrated similar findings [43,47]. Onal et al.’s investigation of outcomes in esophageal cancer patients with isolated synchronous brain oligometastasis included five patients with adenocarcinoma out of the seven included for analysis [48]. Patients underwent definitive chemoradiotherapy of the primary tumor and locally ablative treatment of the brain metastasis, with a median time to progression of 8 months and median survival of 18.9 months. The nuance and outcome variations of these relatively small retrospective cohort studies demonstrate the need for larger randomized control trials in this cohort. Additionally, treatment options should be stratified based on the timing of oligometastasis presentation and location, as these factors appear to affect survival.

Outside of surgical resection, the role of stereotactic body radiation therapy (SBRT) and radiofrequency ablation for oligometastasis has also been recently explored, though reports are limited. Inderson et al.’s report an endoscopic ultrasound guided radiofrequency ablation for left adrenal oligometastasis following EA with acceptable outcome [49]. Pulmonary oligometastases treated with SBRT also appear to be safe and feasible for local control with minimal toxicity [50]. Larger SBRT studies that include synchronous metastasis or oligorecurrence of esophageal carcinoma have demonstrated the safety and efficacy of this treatment strategy [51]. Unfortunately, there are no randomized trials on salvage treatment modalities for oligometastasis in this context and no large comparisons between resection, SBRT, radiofrequency ablation, or alternative treatment methods. However, locally ablative or surgical resection approaches do appear to confirm some survival benefit. Further work to elucidate the preferred treatment strategies are needed. The “best” approach is likely one that is tailored to the individual patient and accounts for timing of oligometastatic presentation, metastatic location, size, and patient status.

**Figure 2:**
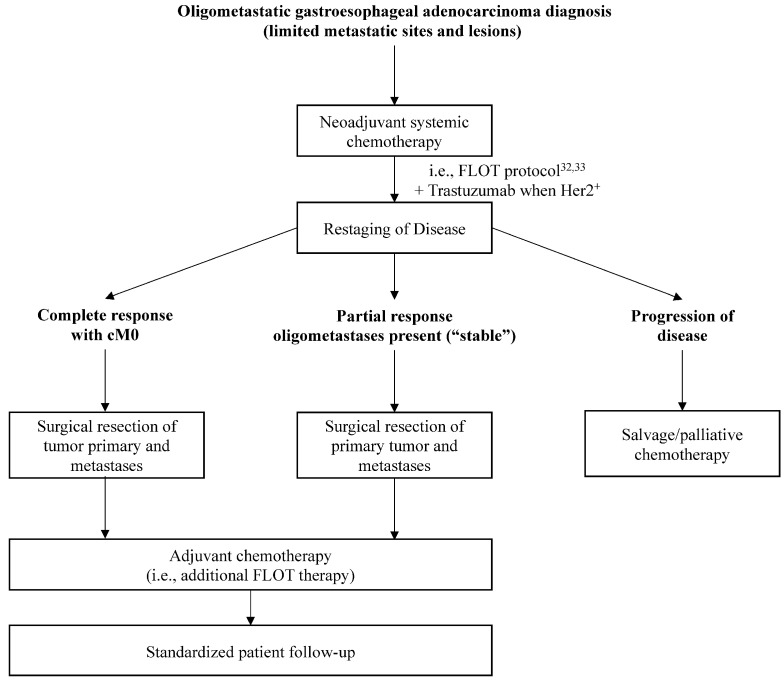
Treatment algorithm for patients with oligometastatic gastroesophageal carcinoma as proposed by Schmidt et al. [45]. Individual patients should additionally be discussed in the context of a multi-disciplinary tumor board. FLOT protocol includes: 5-Fluorouracil, Leucovorin, Oxaliplatin, and Docetaxel [31].

The prospective multicenter phase 2 AIO-FLOT3 trial evaluated 252 GEJ or gastric adenocarcinoma patients (116 gastroesophageal junction, 152 gastric) in three-arms: (a) primarily operable tumors, (b) limited metastatic patients, and (c) diffusely metastatic patients [31]. Patients in all arms received FLOT protocol chemotherapy (5-fluorouracil, leucovorin, oxalioplatin, and docetaxel), with those in arm B receiving four cycles with subsequent restaging with computed tomography and magnetic resonance imaging. If R0 resection of the primary tumor and at least macroscopic complete resection of metastases was felt possible, patients received an additional four cycles of FLOT, followed by surgical resection. Of the available 60 patients evaluated in arm B, 45% of patients had retroperitoneal lymph node metastases, 18.3% liver metastases, 16.7% lung metastases, 6.7% localized peritoneal involvement, and 13.3% other sites [31]. Patients receiving surgical resection within arm B had significantly longer overall survival (31.3 months) than those who did not undergo resection (15.9 months). The response rate for patients in arm B was also higher than those in arm C. On the basis of this trial, Schmidt et al. proposed a potential treatment algorithm for patients with gastroesophageal carcinoma with synchronous oligometastasis in consultation with a multidisciplinary tumor board (Figure 2) [45]. While locally advanced, operable patients represent a distinct cohort compared to those with widely metastatic disease, the overall survival of patients remains notable.

The majority of existing data for management of oligometastatic disease are retrospective in nature and therefore limited in application due to heterogeneity of tumor classification and treatment modalities within studies. On the basis of the existing evidence, aggressive therapy with metastasectomy appears superior to palliative chemotherapy alone in select patients [41,42,45]. Further, neoadjuvant/peri-operative FLOT therapy should be recommended to all patients in this cohort, given the prognosis improvement over other regimens [31,32]. Management of synchronous or metachronous oligometastasis with surgical metastatectomy or ablative/SBRT treatment strategies should likely be individually tailored, with consideration of prior history of surgical intervention to the affected area, likelihood of prolonged meaningful survival or potential cure, and minimization of complications. Treatment with immunotherapy in patients with PD-L1 mutations and the addition of trastuzumab for Her2+ patients should also be considered in the context of multi-modal therapy. Due to the paucity of available randomized trial data, it remains unclear whether an aggressive surgical approach in the case of limited metastasis prolongs patient survival or whether available survival results are influenced by patient selection. Nonetheless, patients with oligometastatic disease should be discussed in the context of a multi-disciplinary tumor board as part of an individualized treatment approach with enrollment as part of a study, if able. Additionally, the importance of surgical resection as part of multimodal therapy in locally metastatic esophageal adenocarcinoma cannot be ignored. Guidance from the upcoming RENAISSANCE AIO-FLOT5 trial is anxiously anticipated and will likely result in an update to existing guidelines.

### 3.5. Future Projections

Given the paucity of large randomized control trial data in this cohort, practice changing guidelines based on the available evidence is indeed difficult. The aforementioned prospective multicenter randomized RENIASSANE (AIO-FLOT5) trial aims to investigate the potential role of surgical intervention in oligometastatic GEJ and gastric carcinoma [34]. The trial will allocate 271 total patients into two arms: those presenting with limited metastatic stage (defined in the trial as retroperitoneal lymph node metastases only or a maximum of one incurable organ site that is potentially resectable or locally controllable with or without retroperitoneal lymph nodes) will receive 4 cycles of FLOT chemotherapy or trastuzumab if Her2+. Those without disease progression will then be randomized 1:1 to receive additional chemotherapy or surgical resection of the primary tumor and metastases followed by chemotherapy. This trial is suited to lead to practice changing guidelines, either advocating for surgical resection or, alternatively, excluding these select patients from consideration of surgical intervention.

While foregut surgeons and oncologists await the results of the impact of surgical intervention, immunotherapy is quickly becoming part of the treatment regimen in GEJ carcinoma. Recently presented results of the CheckMate 577 and KEYNOTE-590 trials demonstrated encouraging results and have impacted current treatment guidelines in this particular cohort [52,53]. KEYNOTE-590 included 749 patients with locally advanced or metastatic adenocarcinoma or esophageal squamous cell carcinoma or Siewert type 1 esophagogastric junction adenocarcinoma, who were randomized to receive either pembrolizumab plus 5-FU and cisplatin or placebo plus this chemotherapeutic regimen [53]. Overall, pembrolizumab with chemotherapy resulted in a median survival of 12.4 months compared to 9.8 months in the placebo arm. Additionally, patients with high levels of PD-L1 had a median survival of 13.5 months versus 9.4 months compared to placebo with chemotherapy [53]. Similarly, the recently published results of the multi-center, randomized, double-blind CheckMate-577 trial demonstrated superior results of adjuvant nivolumab in patients with resected esophageal or esophagogastric junction cancer who had received neoadjuvant chemoradiotherapy and R0 resection with residual pathologic disease (22.4 months vs. 11 months) [52].

These trials highlight the active investigation into novel treatment modalities and emerging practice-changing advances in esophageal and gastric carcinoma. Further research into tumor microenvironment, molecular alterations, gene expression, and surgical techniques will give way to innovative treatment modalities including advanced chemotherapeutics, targeted therapies, and advanced surgical approaches.

## 4. Conclusions

The support of surgical intervention in oligometastatic esophageal adenocarcinoma has continued to gain favor over the last decade in carefully selected patients [31,35,36,37,38,39,40,41,42,43]. Emerging randomized trial evidence is set to define this role and quantify the potential benefit of surgical resection, or lack thereof. Important innovations in chemotherapeutics and targeted therapies are currently reimagining treatment paradigms. The importance of an experienced multidisciplinary team approach and tailored treatment strategy cannot be understated. Overall, patient selection remains paramount to ensuring optimal outcomes and should include consideration for resectability of the primary tumor and metastases, general patient condition, and response to chemotherapy.

## References

[1] Napier K.J., Scheerer M., Misra S. (2014). Esophageal cancer: A Review of epidemiology, pathogenesis, staging workup and treatment modalities. World J. Gastrointest. Oncol..

[2] Derakhshan M.H., Arnold M., Brewster D.H., Going J.J., Mitchell D.R., Forman D., McColl K.E. (2016). Worldwide Inverse Association between Gastric Cancer and Esophageal Adenocarcinoma Suggesting a Common Environmental Factor Exerting Opposing Effects. Am. J. Gastroenterol..

[3] Howlader N.N.A., Krapcho M., Miller D., Brest A., Yu M., Ruhl J., Tatalovich Z., Mariotto A., Lewis D.R., Chen H.S. (2021). SEER Cancer Statistics Review, 1975–2018.

[4] Medical Research Council Oesophageal Cancer Working G. (2002). Surgical resection with or without preoperative chemotherapy in oesophageal cancer: A randomised controlled trial. Lancet.

[5] Lee D.H., Kim H.R., Kim D.K., Park S.I., Kim Y.H. (2013). Outcomes of cervical lymph node recurrence in patients with esophageal squamous cell carcinoma after esophagectomy with 2-field lymph node dissection. J. Thorac. Cardiovasc. Surg..

[6] Abate E., DeMeester S.R., Zehetner J., Oezcelik A., Ayazi S., Costales J., Banki F., Lipham J.C., Hagen J.A. (2010). DeMeester TR. Recurrence after esophagectomy for adenocarcinoma: Defining optimal follow-up intervals and testing. J. Am. Coll. Surg..

[7] Shapiro J., van Lanschot J.J.B., Hulshof M., van Hagen P., van Berge Henegouwen M.I., Wijnhoven B.P.L., van Laarhoven H.W.M., Nieuwenhuijzen G.A.P., Hospers G.A.P., Bonenkamp J.J. (2015). Neoadjuvant chemoradiotherapy plus surgery versus surgery alone for oesophageal or junctional cancer (CROSS): Long-term results of a randomised controlled trial. Lancet Oncol..

[8] Tepper J., Krasna M.J., Niedzwiecki D., Hollis D., Reed C.E., Goldberg R., Kiel K., Willett C., Sugarbaker D., Mayer R. (2008). Phase III trial of trimodality therapy with cisplatin, fluorouracil, radiotherapy, and surgery compared with surgery alone for esophageal cancer: CALGB 9781. J. Clin. Oncol..

[9] Jamel S., Tukanova K., Markar S. (2019). Detection and management of oligometastatic disease in oesophageal cancer and identification of prognostic factors: A systematic review. World J. Gastrointest. Oncol..

[10] Weichselbaum R.R., Hellman S. (2011). Oligometastases revisited. Nat. Rev. Clin. Oncol..

[11] Reyes D.K., Pienta K.J. (2015). The biology and treatment of oligometastatic cancer. Oncotarget.

[12] Varghese T.K., Hofstetter W.L., Rizk N.P., Low D.E., Darling G.E., Watson T.J., Mitchell J.D., Krasna M.J. (2013). The society of thoracic surgeons guidelines on the diagnosis and staging of patients with esophageal cancer. Ann. Thorac. Surg..

[13] Vazquez-Sequeiros E., Norton I.D., Clain J.E., Wang K.K., Affi A., Allen M., Deschamps C., Miller D., Salomao D., Wiersema M.J. (2001). Impact of EUS-guided fine-needle aspiration on lymph node staging in patients with esophageal carcinoma. Gastrointest. Endosc..

[14] Saab J., Zia H., Mathew S., Kluk M., Narula N., Fernandes H. (2017). Utility of Genomic Analysis in Differentiating Synchronous and Metachronous Lung Adenocarcinomas from Primary Adenocarcinomas with Intrapulmonary Metastasis. Transl. Oncol..

[15] Suzuki A., Xiao L., Hayashi Y., Macapinlac H.A., Welsh J., Lin S.H., Lee J.H., Bhutani M.S., Maru D.M., Hofstetter W.L. (2011). Prognostic significance of baseline positron emission tomography and importance of clinical complete response in patients with esophageal or gastroesophageal junction cancer treated with definitive chemoradiotherapy. Cancer.

[16] NCCN Clinical Practice Guidelines in Oncology: Esophageal and Esophagogastric Junction Cancers. http://www.nccn.org/professionals/physician_gls/pdf/esophageal.pdf.

[17] Testa U., Castelli G., Pelosi E. (2017). Esophageal Cancer: Genomic and Molecular Characterization, Stem Cell Compartment and Clonal Evolution. Medicines.

[18] Dulak A.M., Stojanov P., Peng S., Lawrence M.S., Fox C., Stewart C., Bandla S., Imamura Y., Schumacher S.E., Shefler E. (2013). Exome and whole-genome sequencing of esophageal adenocarcinoma identifies recurrent driver events and mutational complexity. Nat. Genet..

[19] Wang Z., Lin L., Thomas D.G., Nadal E., Chang A.C., Beer D.G., Lin J. (2015). The role of Dickkopf-3 overexpression in esophageal adenocarcinoma. J. Thorac. Cardiovasc. Surg..

[20] Helminen O., Huhta H., Leppanen J., Kauppila J.H., Takala H., Lehenkari P.P., Saarnio J., Karttunen T.J. (2016). Nuclear localization of Toll-like receptor 5 in Barrett’s esophagus and esophageal adenocarcinoma is associated with metastatic behavior. Virchows Arch..

[21] Xu Y., Chen Y., Wei L., Lai S., Zheng W., Wu F. (2019). Serum tumor-associated glycoprotein 72, a helpful predictor of lymph nodes invasion in esophagogastric junction adenocarcinoma. Biochem. Biophys. Res. Commun..

[22] Jung J.O., Nienhuser H., Schleussner N., Schmidt T. (2020). Oligometastatic Gastroesophageal Adenocarcinoma: Molecular Pathophysiology and Current Therapeutic Approach. Int. J. Mol. Sci..

[23] Castano-Rodriguez N., Kaakoush N.O., Mitchell H.M. (2014). Pattern-recognition receptors and gastric cancer. Front Immunol..

[24] Barghash A., Golob-Schwarzl N., Helms V., Haybaeck J., Kessler S.M. (2016). Elevated expression of the IGF2 mRNA binding protein 2 (IGF2BP2/IMP2) is linked to short survival and metastasis in esophageal adenocarcinoma. Oncotarget.

[25] Haussler U., Bitzer M., Bosmuller H., Clasen S., Gotz M., Malek N.P., Plentz R.R. (2016). AFP-producing adenocarcinoma of the esophagogastric junction: Report of a case with atypical immunohistochemical findings responding to palliative chemotherapy with 5-fluorouracil, leucovorin, oxaliplatin, and docetaxel (FLOT regime). Z. Gastroenterol..

[26] Miyazaki T., Sohda M., Sakai M., Kumakura Y., Yoshida T., Kuriyama K., Yokobori T., Miyazaki M., Hirato J., Okumura T. (2018). Multimodality Therapy Including Proton Beam Therapy for AFP Producing Esophageal Cancer with Multiple Liver Metastases. Intern. Med..

[27] Nagai Y., Kato T., Harano M., Satoh D., Choda Y., Tokumoto N., Kanazawa T., Matsukawa H., Ojima Y., Idani H. (2014). [A case of AFP-producing esophagogastric junction cancer with liver metastases with a good response to chemotherapy]. Gan Kagaku Ryoho.

[28] Siewert J.R., Stein H.J. (1996). Carcinoma of the gastroesophageal junction—classification, pathology and extent of resection. Dis. Esophagus.

[29] Lagergren J., Smyth E., Cunningham D., Lagergren P. (2017). Oesophageal cancer. Lancet.

[30] Herskovic A., Martz K., al-Sarraf M., Leichman L., Brindle J., Vaitkevicius V., Cooper J., Byhardt R., Davis L., Emami B. (1992). Combined chemotherapy and radiotherapy compared with radiotherapy alone in patients with cancer of the esophagus. N. Engl. J. Med..

[31] Al-Batran S.E., Homann N., Pauligk C., Illerhaus G., Martens U.M., Stoehlmacher J., Schmalenberg H., Luley K.B., Prasnikar N., Egger M. (2017). Effect of Neoadjuvant Chemotherapy Followed by Surgical Resection on Survival in Patients with Limited Metastatic Gastric or Gastroesophageal Junction Cancer: The AIO-FLOT3 Trial. JAMA Oncol..

[32] Al-Batran S.E., Homann N., Pauligk C., Goetze T.O., Meiler J., Kasper S., Kopp H.G., Mayer F., Haag G.M., Luley K. (2019). Perioperative chemotherapy with fluorouracil plus leucovorin, oxaliplatin, and docetaxel versus fluorouracil or capecitabine plus cisplatin and epirubicin for locally advanced, resectable gastric or gastro-oesophageal junction adenocarcinoma (FLOT4): A randomised, phase 2/3 trial. Lancet.

[33] Yoshida M., Ohtsu A., Boku N., Miyata Y., Shirao K., Shimada Y., Hyodo I., Koizumi W., Kurihara M., Yoshida S. (2004). Long-term survival and prognostic factors in patients with metastatic gastric cancers treated with chemotherapy in the Japan Clinical Oncology Group (JCOG) study. Jpn. J. Clin. Oncol..

[34] Al-Batran S.E., Goetze T.O., Mueller D.W., Vogel A., Winkler M., Lorenzen S., Novotny A., Pauligk C., Homann N., Jungbluth T. (2017). The RENAISSANCE (AIO-FLOT5) trial: Effect of chemotherapy alone vs. chemotherapy followed by surgical resection on survival and quality of life in patients with limited-metastatic adenocarcinoma of the stomach or esophagogastric junction—a phase III trial of the German AIO/CAO-V/CAOGI. BMC Cancer.

[35] Chen F., Sato K., Sakai H., Miyahara R., Bando T., Okubo K., Hirata T. (2008). Date H Pulmonary resection for metastasis from esophageal carcinoma. Interact Cardiovasc. Thorac. Surg..

[36] Ghaly G., Harrison S., Kamel M.K., Rahouma M., Nasar A., Port J.L., Stiles B.M., Altorki N.K. (2018). Predictors of Survival After Treatment of Oligometastases After Esophagectomy. Ann. Thorac. Surg..

[37] Shiono S., Kawamura M., Sato T., Nakagawa K., Nakajima J., Yoshino I., Ikeda N., Horio H., Akiyama H., Kobayashi K. (2008). Disease-free interval length correlates to prognosis of patients who underwent metastasectomy for esophageal lung metastases. J. Thorac. Oncol..

[38] Kozu Y., Sato H., Tsubosa Y., Ogawa H., Yasui H., Kondo H. (2011). Surgical treatment for pulmonary metastases from esophageal carcinoma after definitive chemoradiotherapy: Experience from a single institution. J. Cardiothorac. Surg..

[39] Ichikawa H., Kosugi S., Nakagawa S., Kanda T., Tsuchida M., Koike T., Tanaka O., Hatakeyama K. (2011). Operative treatment for metachronous pulmonary metastasis from esophageal carcinoma. Surgery.

[40] Takemura M., Sakurai K., Takii M., Yoshida K. (2012). Metachronous pulmonary metastasis after radical esophagectomy for esophageal cancer: Prognosis and outcome. J. Cardiothorac. Surg..

[41] Schmidt T., Alldinger I., Blank S., Klose J., Springfeld C., Dreikhausen L., Weichert W., Grenacher L., Bruckner T., Lordick F. (2015). Surgery in oesophago-gastric cancer with metastatic disease: Treatment, prognosis and preoperative patient selection. Eur. J. Surg. Oncol..

[42] Ichida H., Imamura H., Yoshimoto J., Sugo H., Kajiyama Y., Tsurumaru M., Suzuki K., Ishizaki Y., Kawasaki S. (2013). Pattern of postoperative recurrence and hepatic and/or pulmonary resection for liver and/or lung metastases from esophageal carcinoma. World J. Surg..

[43] Huddy J.R., Thomas R.L., Worthington T.R., Karanjia N.D. (2015). Liver metastases from esophageal carcinoma: Is there a role for surgical resection?. Dis. Esophagus..

[44] Schizas D., Mylonas K.S., Kapsampelis P., Bagias G., Katsaros I., Frountzas M., Hemmati P., Liakakos T. (2020). Patients undergoing surgery for oligometastatic oesophageal cancer survive for more than 2 years: Bootstrapping systematic review data. Interact Cardiovasc. Thorac. Surg..

[45] Schmidt T., Monig S.P. (2017). Therapeutic approach in oligometastatic gastric and esophageal cancer. Chirurg.

[46] Nakagawa S., Nishimaki T., Kosugi S., Ohashi M., Kanda T., Hatakeyama K. (2003). Cervical lymphadenectomy is beneficial for patients with carcinoma of the upper and mid-thoracic esophagus. Dis. Esophagus..

[47] Hiyoshi Y., Morita M., Kawano H., Otsu H., Ando K., Ito S., Miyamoto Y., Sakamoto Y., Saeki H., Oki E. (2015). Clinical significance of surgical resection for the recurrence of esophageal cancer after radical esophagectomy. Ann. Surg. Oncol..

[48] Onal C., Akkus Yildirim B., Guler O.C. (2017). Outcomes of aggressive treatment in esophageal cancer patients with synchronous solitary brain metastasis. Mol. Clin. Oncol..

[49] Inderson A., Slingerland M., Farina Sarasqueta A., de Steur W.O., Boonstra J.J. (2018). EUS-guided radiofrequency ablation for a left adrenal oligometastasis of an esophageal adenocarcinoma. VideoGIE.

[50] Chai G., Yin Y., Zhou X., Hu Q., Lv B., Li Z., Shi M., Zhao L. (2020). Pulmonary oligometastases treated by stereotactic body radiation therapy (SBRT): A single institution’s experience. Transl. Lung Cancer Res..

[51] Wang H.H., Zaorsky N.G., Meng M.B., Zeng X.L., Deng L., Song Y.C., Zhuang H.Q., Li F.T., Zhao L.J., Yuan Z.Y. (2016). Stereotactic radiation therapy for oligometastases or oligorecurrence within mediastinal lymph nodes. Oncotarget.

[52] Kelly R.J., Ajani J.A., Kuzdzal J., Zander T., Van Cutsem E., Piessen G., Mendez G., Feliciano J., Motoyama S., Lievre A. (2021). Adjuvant Nivolumab in Resected Esophageal or Gastroesophageal Junction Cancer. N. Engl. J. Med..

[53] Kato K., Sun J.-M., Shah M., Enzinger P., Adenis A., Doi T., Kojima T., Metges J.-P., Li Z., Kim S.-B. (2020). Pembrolizumab Plus Chemotherapy Versus Chemotherapy as First-Line Therapy in Patients with Advanced Esophageal Cancer: The Phase 3 KEYNOTE-590 Study. Ann. Oncol..

